# Autophagy-dependent regulation of tumor metastasis by myeloid cells

**DOI:** 10.1371/journal.pone.0179357

**Published:** 2017-07-07

**Authors:** Masahisa Jinushi, Tomoko Morita, Zhihang Xu, Ichiro Kinoshita, Hirotoshi Dosaka-Akita, Hideo Yagita, Yutaka Kawakami

**Affiliations:** 1 Division of Cellular Signaling, Institute for Advanced Medical Research, Keio University School of Medicine, Tokyo, Japan; 2 Institute for Genetic Medicine, Hokkaido University, Sapporo, Japan; 3 Acupuncture and Moxibustion College of Tianjin, University of Traditional Chinese Medicine, TianJin, China; 4 Department of Medical Oncology, Hokkaido University Graduate School of Medicine, Sapporo, Japan; 5 Department of Immunology, Juntendo University School of Medicine, Tokyo, Japan; National Cancer Institute, UNITED STATES

## Abstract

Autophagy is a vital process controlling the lysosomal degradation of cellular organelles and thereby regulating tissue homeostasis in an environment-dependent fashion. Recent studies have unveiled the critical role of tumor cell-derived autophagy in regulating pro-tumor and anti-tumor processes depending on different stages and tumor microenvironments. However, the precise mechanism whereby autophagy regulates tumor progression remains largely unclear. Since myeloid cells contribute to tumor progression and metastasis, we evaluated the role of myeloid cell-specific autophagy in the regulation of tumor progression. We found that the number and size of metastatic lesions were smaller in myeloid cell-specific autophagy-deficient mice. Furthermore, autophagy-mediated regulation of TGF-β in myeloid cells was associated with the induction of epithelial-mesenchymal transition (EMT), which increases the invasive and metastatic potentials of tumor cells. Myeloid-derived autophagy also plays a critical role in impairing antitumor immune responses and promoting the survival and accumulation of M2 macrophages in tumor tissues in a CSF-1 and TGF-β-dependent manner. Taken together, our findings elucidate previously unrecognized mechanisms by which myeloid cells promote tumor progression through autophagy-mediated regulation of malignancy and immune tolerance.

## Introduction

Tumor microenvironments (TME) regulate the tumorigenic activities of transformed cells in coordination with multiple tumor-infiltrating normal cells such as endothelial cells, fibroblasts, mesenchymal stem cells and inflammatory cells [1,2]. In particular, recent studies have revealed the importance of tumor-associated myeloid cells (TAM) in tumor progression. TAM support tumor progression through various mechanisms including tumor angiogenesis, immune suppression, matrix remodeling and the epithelial-mesenchymal transition (EMT) of malignant cells [3,4]. Thus, the detailed evaluation of molecular mechanisms that govern the complex interplay between TAM and transformed cells must be defined in order to control the dismal clinical course of malignancy and improve patient responsiveness to anticancer therapeutics.

Autophagy is a vital physiological pathway that maintains metabolic homeostasis and controls stress responses by capturing intra- and extra-cellular components in autophagic vesicles and processing them in the lysosomal degradation system [5,6]. While accumulating evidence has clarified the contribution of autophagy to tumor initiation and progression, it has been proposed that autophagic signals in tumor cells either promote or suppress tumor growth in a context-dependent manner [7–10]. Deficiency of autophagy-essential genes, such as Beclin-1 and Atg8, increased tumorigenicity, and autophagy protects cells from transformation through protection excess oxidative stress in p62-dependent manner [7–9]. On the other hands, Ras utilize autophagy to facilitate lung tumorigenicity by modulating several metabolic pathway [10,11]. Thus, the mechanism by which autophagic pathways in tumor-infiltrating non-transformed cells regulate tumorigenicity in a TME-dependent manner remains elusive.

In this study, we demonstrate the unique role of myeloid cell autophagic pathways in the regulation of the malignant properties of tumor cells. Although myeloid cell-derived autophagy is dispensable for subcutaneous tumor growth, it facilitates the invasive and metastatic properties of tumor cells through TGF-β1-mediated induction of EMT and immune tolerance. Moreover, myeloid cell-derived autophagy contributes to the enhanced survival in stressed TME and the differentiation of M2-like macrophages induced by tumor-derived colony-stimulating factor-1 (CSF-1). Our findings reveal a new biological aspect of myeloid cell-derived autophagy in supporting tumor metastasis and progression.

## Materials and methods

### Mice

C57BL/6 and BALB/c mice were purchased from SCL. MMTV-PyMT mice were purchased from Jackson Laboratory. Atg5^flox/flox^, Lysozome M (LysM)-Cre/Atg5^flox/flox^ (LysM-Atg5^-/-^) and OT-I mice were used as described previously [12,13]. All experiments were conducted under a protocol approved by the animal care committees of Hokkaido University, and all mice were cared ethically and strictly followed the declaration of Helsinki with proper Housing and husbandry environment. We were monitored at least once a week of all animal health conditions, and there were no case that severely ill or died at anytime prior to the experimental endpoint. We followed the protocol recommended by our institute for early euthanasia/humane endpoints for animals. CD11b-positive myeloid cells were purified by anti-CD11b-labelled microbeads (Miltenyi Biotech) from protease-digested tumor tissues.

### Patient samples

The clinical protocols for this study were approved by the committees in the Institutional Review Board of Hokkaido University Hospital (Approval number: 10–0114). CD11b^+^ cells were obtained from the tumors or peripheral blood of patients with stage IV non-small cell lung carcinomas after written informed consent had been obtained. The cells were isolated by Ficoll-Hypaque density centrifugation, and further purified by anti-CD11b-labelled microbeads (Miltenyi Biotech).

### Tumor cells

Tumor cells (B16-F10 melanoma & MC38 colon carcinoma) were obtained from the American Type Culture Collection (ATCC). All cell lines were obtained one year before being used in experiments and authenticated by the Central Institute for Experimental Animals (Kawasaki, Japan) for interspecies and mycoplasma contamination by PCR within 3 months of the experiments.

### In vivo tumorigenic assays

C57BL/6 wild-type, LysM-Atg5^-/-^ or Atg5^flox/flox^ mice were injected subcutaneously in the flank with 1x10^5^ B16-F10 or MC38 cells. Tumor growth was measured on the indicated days. For metastatic assays, B16-F10 or MC38 tumor cells were injected intravenously and intraperitoneally into LysM-Atg5^-/-^ or Atg5^flox/flox^ mice. The average areas, numbers and weight of lung and liver metastatic lesions were analyzed 3 weeks after the procedures. The average tumor areas were quantified by Image-J software. In some instances, anti-TGF-β1 mAb (1D11; 1 mg/kg per mouse) or CSF-1 receptor kinase inhibitor GW2580 (50 mg/kg) were administered intraperitoneally to the mice twice per week during the experimental period; in other experiments, anti-CD8 mAb (53–6.72, 1 mg/kg) was administered intravenously two days prior to tumor challenge. We confirmed the specificity of GW2580 since it inhibited expression of CSF-1 receptor but not CSF-2 receptor on monocytes.

### In vitro cell viability assay

Atg5^flox/flox^ or LysM-Atg5^-/-^ CD11b^high^TAM were isolated from MC38 or B16-deried tumors and briefly cultured for 16 h. MC38 cells were cultured with 50% of the TAM supernatants for 48 h, and then treated with cisplatin (CDDP) (10 μM) for 48 h. BMDM generated with recombinant CSF-1 (10 μg/mL) for 7 days were cultured under serum-starved conditions or low oxygen concentrations (1%) for 16 h, or exposed to ultraviolet light (300 J/m^2^) for 10 sec and cultured for 16 h. Cell viability was assessed with FITC-labelled annexin V and propidium iodide by flow cytometry. In some experiments, cells were treated with TGF-β receptor kinase inhibitor (SB431542) or blocking antibodies against murine TGF-β1 (Clone 1D11). The specificity of SB431542 was previously confirmed as reported [14].

### In vitro invasion assay

Invasive activities of tumor cells were determined using the BD Bio-Coat Tumor Invasion Assay System (BD Bioscience) according to the manufacturer’s instructions. In brief, the upper chamber was coated with matrigel (100 μl), and serum-free media was used for the bottom chamber. MC38 murine colon cancer cells or A549 human lung cancer cells pretreated with or without myeloid cell supernatants were seeded into the upper chamber. The cells that invaded the bottom chamber were stained with 4 μg/mL calcein AM for 1 h, and then counted using a fluorescence microscope.

### Immunofluorescence microscopy

MC38 cells (1 x 10^4^) stimulated with Atg5^flox/flox^ or LysM-Atg5^-/-^ TAM or recombinant murine TGF-β1 (100 ng/mL) were cultured on a glass chamber plate overnight. The plate was washed three times to remove floating cells and adherent cells were fixed with 20% methanol at –20^°^C for 5 minutes. The cells were stained with antibodies for E-cadherin, N-cadherin, or vimentin and then Alexa-Flow 488- or Cy3-labeled secondary Ab and visualized using a TE2000-U inverted fluorescence microscope (Olympus).

### Cytokine assays

TGF-β1, IL-6, IL-10, IL-12p40 and IFN-γ were measured by ELISA in culture supernatants from TAM and TAN generated from Atg5^flox/flox^ or LysM-Atg5^-/-^ mice, CD11b^+^ cells derived from tumors or peripheral bloods of non-small lung cancer patients according to the manufacturer’s directions (R&D Systems, BD Biosciences).

### mRNA quantification by real-time PCR

The mRNA was isolated from Atg5^flox/flox^ or LysM-Atg5^-/-^ TAM. TGF-β1 forward and reverse primers were 5'-CACCGGAGAGCCCTGGATA-3' and 5'-TGTACAGCTGCCGCACACA—3', respectively. The mRNAs of TGF-β1 were quantified by real-time PCR (RT-PCR) using SYBR Green Gene Expression Assays (Applied Biosystems).

### T cell differentiation assay

T cell differentiation assays were, respectively performed as previously described [15]. In brief, irradiated EG7 tumor cells (1 x 10^6^) were loaded to Atg5^flox/flox^ or LysM-Atg5^-/-^ BMDM (1 x 10^5^) to facilitate phagocytosis and antigen processing. Two hours after the EG7 cells were loaded, they were extensively washed, and then cultured with CD4^+^ or CD8^+^ T cells from wild-type mice for 96 h. Cells were stained with anti-CD3, -CD4, or -CD8 mAbs, fixed and permeabilized with Cytofix / Cytoperm (BD Biosciences). Cells were then further stained with anti-IFN-γ, anti-IL-4, anti-IL-17, anti-Foxp3, anti-granzyme-B and isotype control mAb (BD Biosciences), and analyzed by flow cytometry.

### OVA-specific antitumor immune responses

OVA-specific CD8^+^ T cells isolated from OT-I mice were adoptively transferred (5 x 10^6^ /mice) and two days later EG7 or B16-OVA cells (1 x 10^5^ /mice) were inoculated subcutaneously into C57BL/6 wild-type, LysM-Atg5^-/-^, Atg5^flox/flox^ or bone marrow chimeric mice as described below. The established tumors (~25 mm^2^) were then treated with CDDP (250 μg/mice) in the presence of control Ig or anti-TGF-β1 mAb on days 8, 10 and 12 after tumor inoculation. Four days after the final treatment, tumor-infiltrating myeloid cells were obtained from B16-OVA tumors using density gradient separation. The levels of SIINFEKL/H-2^b^ complex on TAM were analyzed by flow cytometry.

### Statistics

The differences between two groups were determined by the Student’s *t* test or the two-sample *t* test with Welch’s correction. The differences among three or more groups were determined by a one-way ANOVA. *P* values less than 0.05 are considered statistically significant. *: *p<*0.05, ns: not significant.

## Results

### Myeloid-specific autophagy promotes the metastatic potential of tumor cells

**Fig 1:**
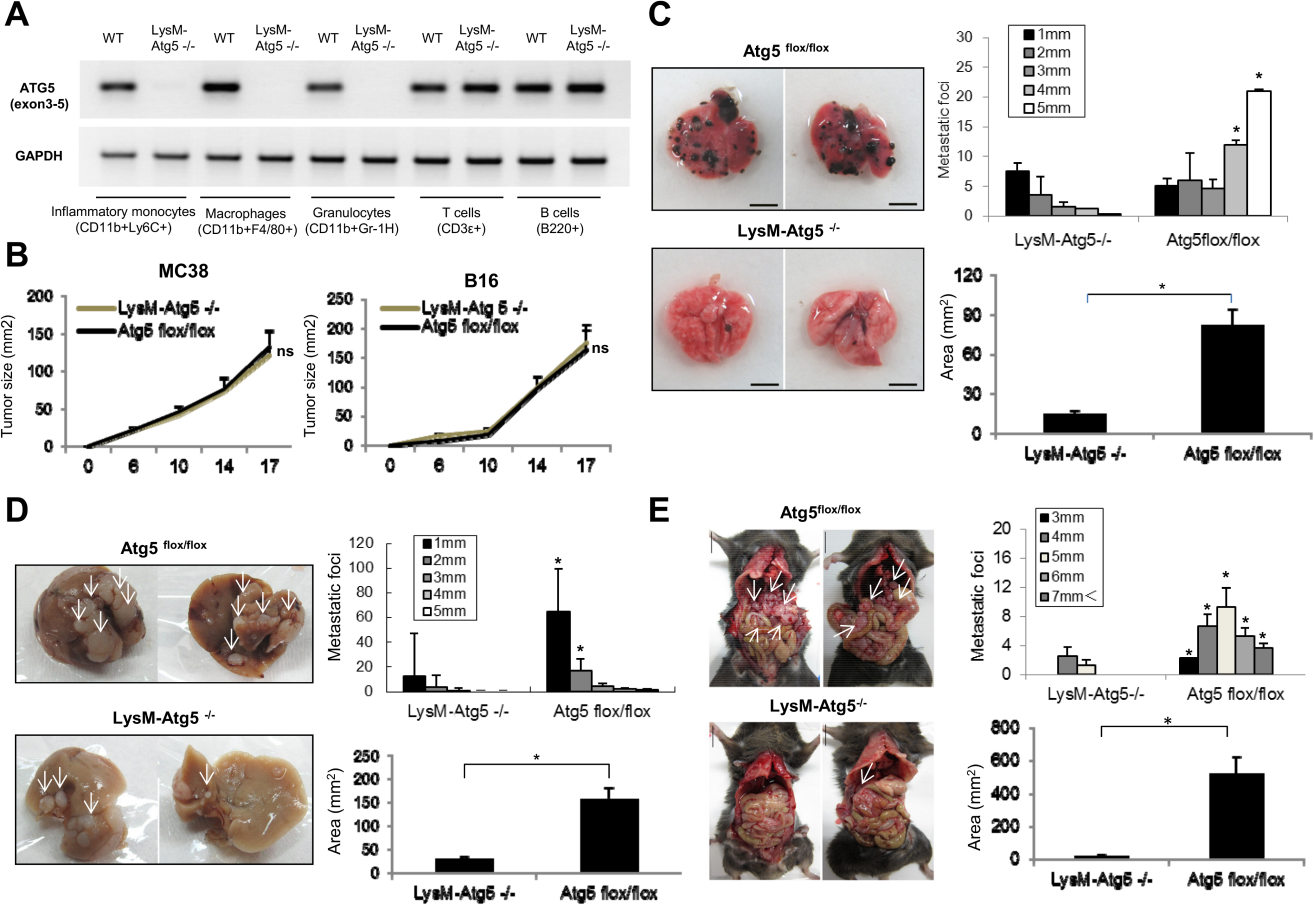
Myeloid cell-specific deletion of autophagy attenuates tumor metastasis. **(A)** The Atg5 gene was specifically deleted in myeloid cells of LysM-Atg5^-/-^ mice. The cells isolated from *in vivo* growing tumors (MC38) were prepared from wild-type (WT) or LysM-Atg5^-/-^ mice, and CD11b^+^Ly6C^+^ monocytes, CD11b^+^F4/80^+^ macrophages, CD11b^+^Gr-1^+^ granulocytes, CD3ε^+^ T cells and B220^+^ B cells were isolated by flow cytometry. Expression of Atg5 was evaluated by RT-PCR. **(B)** Atg5^flox/flox^ or LysM-Atg5 ^-/-^ mice were inoculated subcutaneously with MC38 or B16 cells. Tumor growth was measured on the indicated days. **(C)** B16 cells were injected via tail veins of Atg5^flox/flox^ or LysM-Atg5 ^-/-^ mice. 28 days later, the mice were sacrificed and subjected to macroscopic analysis for metastatic lesions in the lung. The experiments were performed at least ten times with similar results. **(D)** MC38 cells were injected into the spleen of Atg5^flox/flox^ or LysM-Atg5 ^-/-^ mice. Twenty-one days later, the mice were sacrificed and subjected to macroscopic analysis for metastatic lesions in the liver. Representative data (left) and statistical analysis of average numbers and areas (mm^2^) of metastatic lesions are shown (right). The tumor metastatic lesions are shown by white arrows. The experiments were performed at least ten times with similar results. **(E)** MC38 cells were injected into the spleens of Atg5^flox/flox^ or LysM-Atg5 ^-/-^ mice. Twenty-one days later, the mice were sacrificed and subjected to macroscopic analysis for disseminated lesions in the peritoneal cavity. Representative data (left) and statistical analysis of average numbers and areas (mm^2^) of metastatic lesions are shown (right). The experiments were performed five times with similar results. *: p<0.05, ns: not significant.

To characterize the role of myeloid cell autophagic pathways in tumor progression, we generated a myeloid cell-specific targeted deletion of the autophagic gene Atg5 by crossing mice carrying the lox-P flanked Atg5 allele to mice carrying lysozyme M (LysM) promoter-driven Cre recombinase [16]. In this myeloid cell-specific autophagy-deficient model (LysM-Atg5^-/-^), the Atg5 expression was specifically deleted in granulocytes, macrophages and Ly6C^high^ inflammatory monocytes, and the defect of autophagy was confirmed in the LysM-Atg5^-/-^ macrophages under amino acid starvation (Fig 1A). We select Atg5 as representative marker for autophagy function due to its essential role of autophagic processes [17]. We found that subcutaneous tumor growth was comparable in LysM-Atg5^-/-^ and control Atg5^flox/flox^ mice, suggesting that autophagy in myeloid cells made only a minor contribution to primary tumorigenesis (Fig 1B). In contrast, B16 melanomas yielded large and multiple pulmonary metastatic lesions in Atg5^flox/flox^ mice by 28 days after tail vein injection, but the metastatic tumors were fewer and smaller in LysM-Atg5^-/-^ mice (Fig 1C). Furthermore, splenic inoculation of MC38 colon carcinoma cells resulted in decreased numbers of liver metastatic tumors in LysM-Atg5^-/-^ mice compared to their Atg5^flox/flox^ counterparts (Fig 1D). In addition, myeloid cell-specific deletion of Atg5 resulted in the suppression of tumor dissemination to the peritoneal cavity upon splenic injection of MC38 tumor cells (Fig 1E). Splenomegaly by tumor infiltration was detected in Atg5^flox/flox^ control, but not LysM-Atg5^-/-^ mice. Quantitative analysis revealed that the metastasized tumor sizes in LysM-Atg5^-/-^ mice were smaller than those in Atg5^flox/flox^ mice (Fig 1C–1E).

The myeloid cell-specific deletion of Atg7 also manifest the decreased lung metastasis of B16 tumors, implying that myeloid cell-specific autophagy promotes tumor metastasis in general. Taken together, these results demonstrate that the myeloid cell-specific autophagic pathway promotes the invasive and metastatic activities of tumor cells.

### Myeloid-specific autophagy promotes pro-invasive tumor cell activity

**Fig 2:**
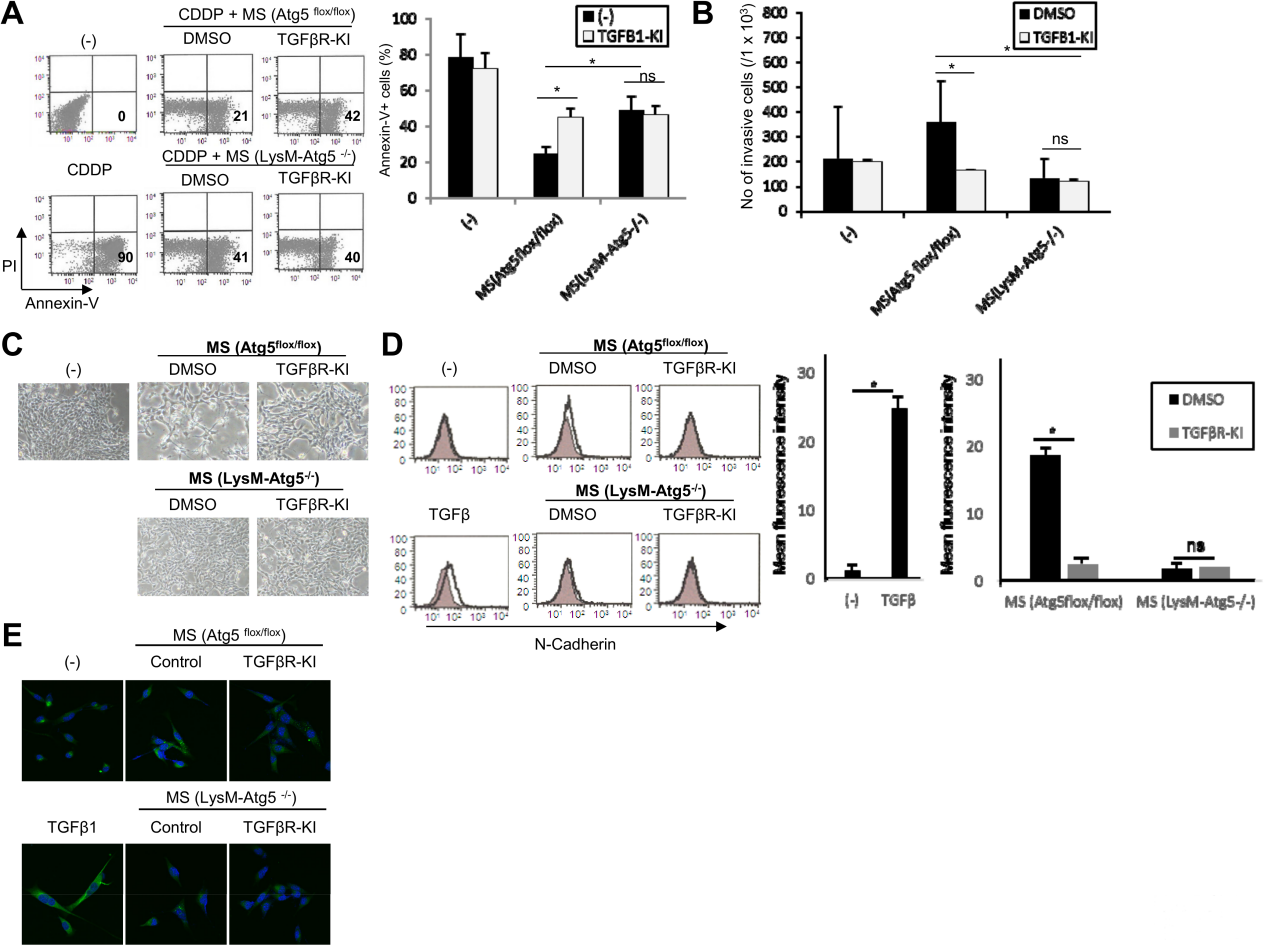
Myeloid cell-specific autophagy supports the chemoresistance and invasive properties of tumor cells through TGF-β1-dependent manner. **(A, B)** MC38 colon cancer cells were cultured with supernatants from Atg5^flox/flox^ or LysM-Atg5^-/-^ TAM (MS) for 48 h in the presence of DMSO or the TGF-β receptor kinase inhibitor SB431542 (TGFβR-KI). **(A)** The indicated cells were then treated with Cisplatin (CDDP: 10 μM) for 24 h, and the cell viability was determined by flow cytometry with PI and Annexin V staining. **(B)** The indicated cells were evaluated for *in vitro* invasion by Matrigel assay. **(C)** MC38 tumor cells were cultured with supernatants from Atg5^flox/flox^ or LysM-Atg5^-/-^ TAM (MS) with or without SB431542 (TGFβR-KI) for 48 h. The cellular morphological appearance was analyzed by microscopy. **(D, E)** MC38 tumor cells were cultured with supernatants from Atg5^flox/flox^ or LysM-Atg5^-/-^ TAM (MS) with or without SB-431542 (TGFβR-KI) or recombinant TGF-β1 (100 ng/mL). The expression levels of N-cadherin (solid line) and vimentin (green) were analyzed by flow cytometry **(D: left)** and confocal microscopy **(E)**, respectively. The statistical analysis was also performed for flow cytometry data (n = 3; D: right). *: p<0.05, ns: not significant.

Autophagic pathways regulate either cellular survival or death in an environment-dependent fashion, but how autophagy in myeloid cells influences cellular homeostasis remains largely unknown. To investigate this, we examined whether myeloid cell-specific autophagy had an impact on tumor cell survival upon induction of stress such as exposure to cytotoxic agents. We isolated TAM from liver and lung metastatic lesions of MC38 and B16 tumors according to CD11b^high^ expression, respectively. CD11b^high^ population generally reflect either activation of inflammatory monocytes, macrophages and granulocytes, and we confirmed mixture of F4/80^high^/Gr-1^low^ and F4/80^low^/Gr-1^high^ subpopulation among CD11b^high^ cells in tumor tissues. There was little difference between each mice on the composition of CD11b^high^F4/80^high^/Gr-1^low^ and CD11b^high^F4/80^low^/Gr-1^high^ in tumor tissues. and the frequencies of TAM populations were similar in both groups. Treatment of supernatants of TAM from wild-type or Atg5^flox/flox^ mice triggered resistance of MC38 tumor cells to cisplatin, as revealed by decreased annexin V and propidium iodide (PI) staining compared to untreated MC38 cells. In contrast, these protective effects were significantly attenuated when supernatants of LysM-Atg5^-/—^derived TAMs were used (Fig 2A). These results underscore a vital contribution of myeloid cell-derived autophagy in supporting the resistance of tumor cells to cytotoxic therapy. We next examined whether myeloid cell autophagy regulates the invasive activities of MC38 tumor cells by *in vitro* matrigel assay. The treatment with supernatant from LysM-Atg5^-/-^ TAM attenuated invasive activities of MC38 tumor cells compared to supernatants from control Atg5^flox/flox^ cells (Fig 2B).

Epithelial-mesenchymal transition (EMT) manifests as a critical step in conferring tumor cells with metastatic activities [18–20]. To investigate whether myeloid cell-specific autophagy regulates morphological and genetic profiles characterized as EMT in tumor cells, we performed confocal microscopy on MC38 tumor cells stimulated with supernatants from LysM-Atg5^-/-^ or Atg5^flox/flox^ TAM. Treatment with supernatants from Atg5^flox/flox^ but not LysM-Atg5^-/-^ TAM caused morphological changes with cobblestone-like epithelial layers of MC38 cells becoming scattered and presenting an elongated fibroblastic shape in appearance (Fig 2C). Furthermore, the tumor cells exhibited robust upregulation of various mesenchymal markers such as vimentin and N-cadherin when stimulated with supernatants from wild-type or Atg5^flox/flox^ TAM (Fig 2D and 2E). In contrast, treatment with supernatant of LysM-Atg5^-/-^ TAM did not induce the EMT-associated changes in MC38 tumor cells. These results demonstrate that autophagy in myeloid cells renders tumor cells with the ability to acquire chemo-resistance and invasive properties by inducing EMT.

### Myeloid autophagy regulates the invasive activities of tumor cells through TGF-β

We found that TGF-β production was positively regulated in tumor-associated myeloid cells in an autophagy-dependent manner. Given critical role of TGF-β in regulating tumorigenicity by promoting anti-cancer drug resistance, metastatic potential and immune suppression [21–24], we next examine whether TGF-β blockade by TGF-β receptor kinase inhibitor (SB431542) modulated the anticancer drug sensitivity and invasive phenotypes of tumor cells stimulated with TAM supernatants. In agreement with our hypothesis, the sensitivity to cisplatin of tumor cells treated with Atg5^flox/flox^ TAM were increased to the levels of those of LysM-Atg5^-/-^ TAM supernatants in the presence of SB431542 (Fig 2A). On the other hands, the sensitivity to cisplatin and invasive activities of tumor cells treated with Atg5^flox/flox^ TAM were reduced to the levels of those of LysM-Atg5^-/-^ TAM supernatants in the presence of SB431542 (Fig 2B). Treatment with SB431542 partially reversed the epithelial morphology of MC38 tumor cells treated with supernatants of Atg5^flox/flox^ TAM (Fig 2C). Moreover, treatment with SB431542 suppressed the induction of N-cadherin and vimentin, which are closely associated with EMT, in MC38 cells treated with supernatants from Atg5^flox/flox^ TAMs, consistent with previous findings that TGF-β is critical for multiple tumorigenic processes including EMT [21, 22] (Fig 2D and 2E).

**Fig 3:**
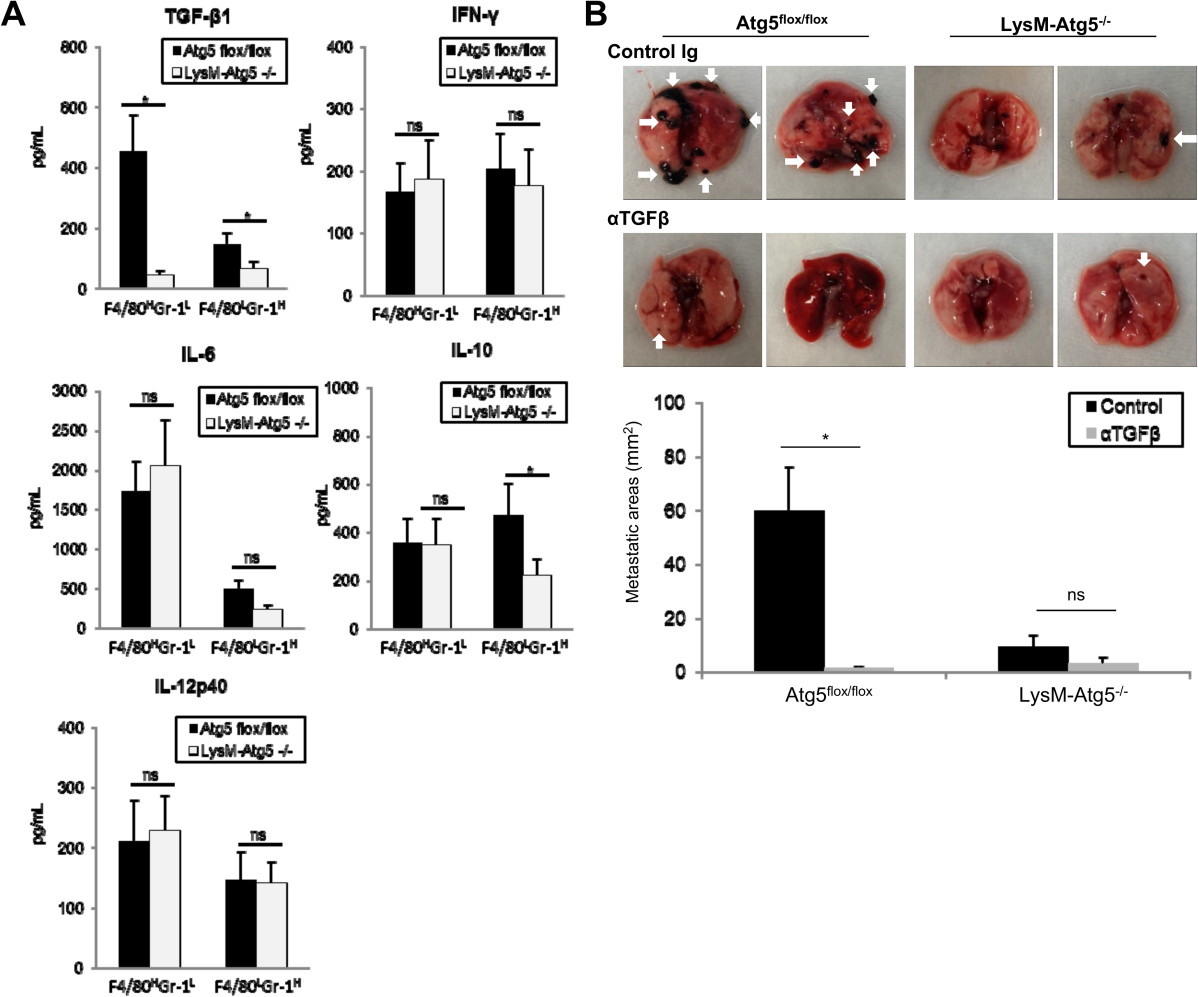
TGF-β upregulated by myeloid cell-specific autophagy is a major mediator in regulating the invasive properties of tumors. **(A)**Production of TGF-β1, IL-6, IL-10, IL-12p40 and IFN-γ was assayed by ELISA in culture supernatants of CD11b^+^F4/80^high^Gr-1^low^(F4/80^H^Gr-1^L^) and CD11b^+^F4/80^low^Gr-1^high^ (F4/80^L^Gr-1^H^) TAM obtained from B16 lung metastasis lesions of Atg5^flox/flox^ or LysM-Atg5^-/-^ mice. **(B)** B16 melanoma cells were injected intravenously into Atg5^flox/flox^ or LysM-Atg5^-/-^ mice. Mice were then treated with anti-TGF-β1 mAb (1D11) or isotype control Ig twice per week. Twenty-one days later, the mice were sacrificed and subjected to macroscopic analysis for metastatic lesions in the lung. The tumor metastatic lesions are shown by white arrows. Representative data (top) and statistical analysis of average areas of metastatic lesions are shown (bottom). *: p<0.05, ns: not significant.

Myeloid cells regulate tumorigenic activities by releasing multiple sets of soluble factors. In addition, accumulating evidence reveals that autophagy contributes to the secretion of multiple cytokines and growth factors [25–27]. We therefore determined whether autophagic signals modulate cytokine profiles in myeloid cells. We found that Atg5^flox/flox^ CD11b^+^F4/80^high^Gr-1^low^ and CD11b^+^F4/80^low^Gr-1^high^ TAM produced TGF-β1 at greater amounts than their LysM-Atg5^-/-^ counterparts (Fig 3A and 3B). In contrast, IL-10 was produced by Atg5^flox/flox^ CD11b^+^F4/80^low^Gr-1^high^ TAM at higher amounts than by their LysM-Atg5^-/-^ counterparts, but was comparable in Atg5^flox/flox^ and LysM-Atg5^-/-^ CD11b^+^F4/80^high^Gr-1^low^TAM. The production of IFN-γ, IL-6 and IL-12p40 was similar in the Atg5^flox/flox^ and LysM-Atg5^-/-^ TAM (Fig 3A). Similarly, myeloid cells of normal splenic and liver macrophages and neutrophils produced comparable levels of these cytokines. We next evaluated whether TGF-β1 regulates the metastatic activities of tumors *in vivo*. Metastatic models of lung and liver cancer using tail vein or splenic administration of B16 or MC38 tumor cells revealed that treatment with anti-TGF-β1 neutralizing antibodies suppressed the formation of macroscopic metastatic foci in Atg5^flox/flox^ mice, whereas it had little effect on metastatic lesions in LysM-Atg5^-/-^ mice (Fig 3B). Overall, TGF-β1 released from myeloid cells in an autophagy-dependent fashion is a critical mediator that promotes tumor cell invasive and metastatic activities.

### Myeloid cell autophagy supports TGF-β production by a translational level

**Fig 4:**
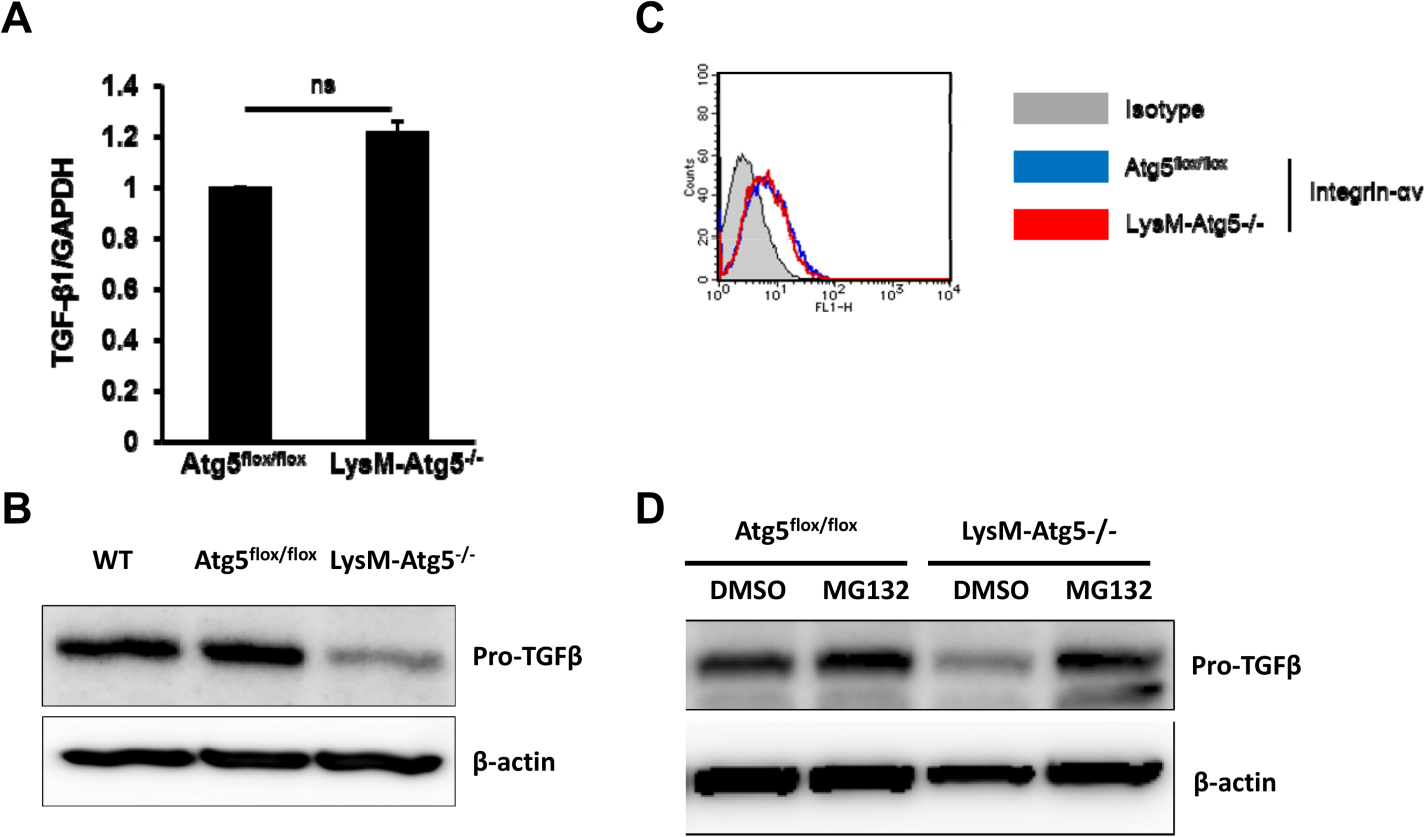
Mechanism of TGF-βinduction by myeloid cell-specific autophagy. **(A)**The mRNA levels of TGF-β1 in Atg5^flox/flox^ or LysM-Atg5^-/-^ TAM were quantified by RT-PCR. **(B)** The protein levels of pro-TGF-β in wild-type (WT), Atg5^flox/flox^ or LysM-Atg5^-/-^ TAM were evaluated by western blot. **(C)** Integrin-αv expression on Atg5^flox/flox^ or LysM-Atg5^-/-^ TAM was evaluated by flow cytometry. **(D)** Atg5^flox/flox^ or LysM-Atg5^-/-^ TAMs were pretreated with MG132 or DMSO for 6h, and pro-TGF-β protein levels were measured by western blot.

To examine mechanisms by which myeloid cells produce TGF-β1 through an autophagy-mediated mechanism, transcriptional and translational regulation of TGF-β1 expression was evaluated in Atg5^flox/flox^ and LysM-Atg5^-/-^ TAM. Although mRNA levels of TGF-β1 were comparable in both, pro-TGF-β1 expression was substantially decreased in LysM-Atg5^-/-^ TAM compared to Atg5^flox/flox^ TAM (Fig 4A and 4B). There was little difference between them on the expression of integrin-αv, which is important to process pro-TGF-β into the active form of TGF-β in myeloid cells (Fig 4A), indicating that autophagy may not be involved in the removal of latency-associated protein (LAP) to release the active form of TGF-β1 [28,29]. Interestingly, autophagy-mediated post-translational regulation of TGF-β might be operated through suppression of proteosomal degradation systems, because the treatment with a proteosomal inhibitor MG132 increased pro-TGF-β expression in LysM-Atg5^-/-^ TAM at similar levels to those in Atg5^flox/flox^ TAM (Fig 4D). Although these results suggest that autophagy supports TGF-β production by suppressing proteosomal degradation of pro-TGF-β in TAM, further study will be required for clarifying the precise mechanisms how myeloid cell-derived autophagy regulates TGF-β protein synthesis.

### Myeloid cell autophagy attenuates antitumor immune responses

**Fig 5:**
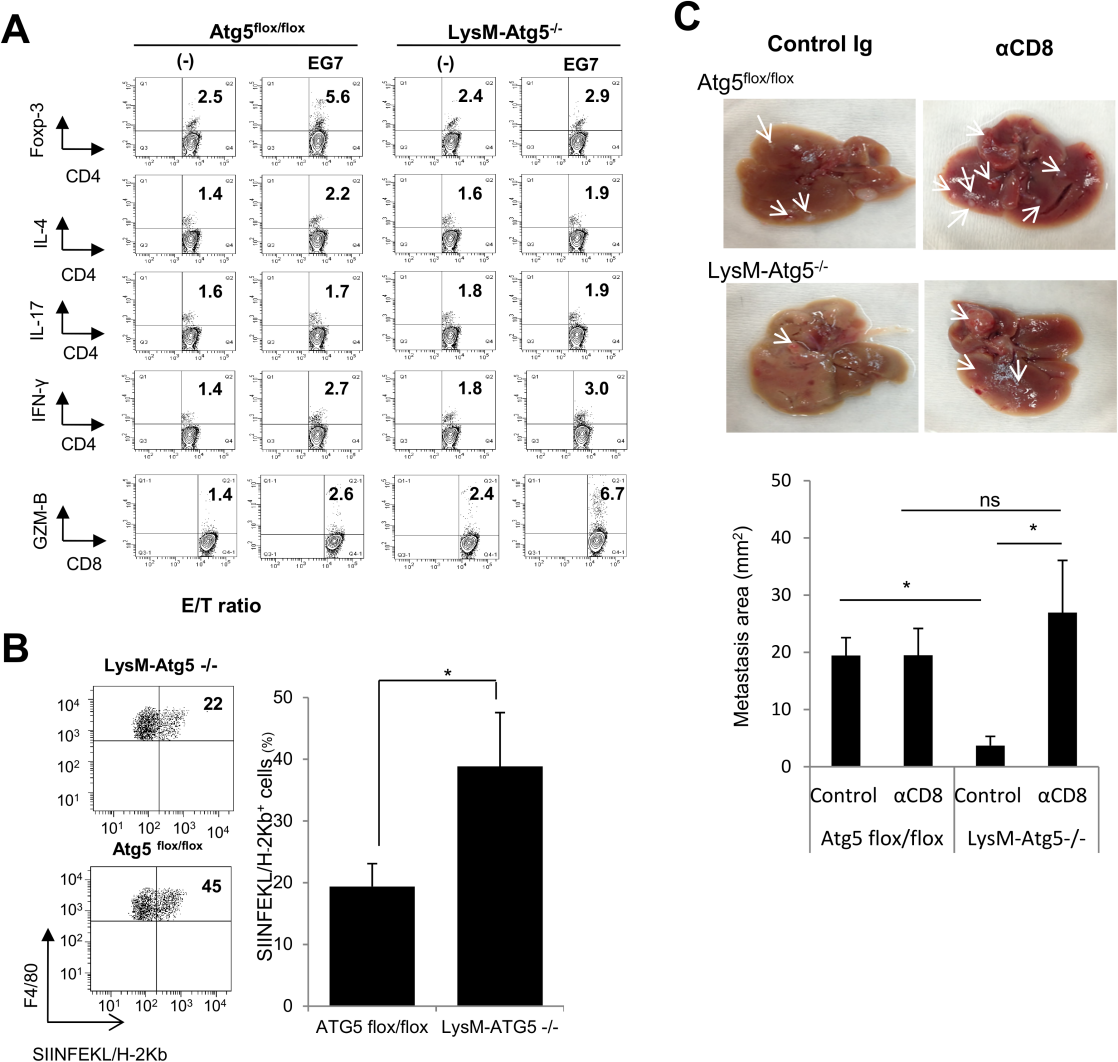
Intratumoral immunomodulation by myeloid cell-specific autophagy. **(A)** Atg5^flox/flox^ or LysM-Atg5 ^-/-^ BMDM were exposed to irradiated EG7 cells for 2 h and then co-cultured with wild-type CD4^+^ or CD8^+^ T cells for 96 h. CD4^+^ T cells expressing IFN-γ, IL-4, IL-17 or Foxp3, as well as CD8^+^ T cells expressing granzyme-B (GZM-B) were assayed by flow cytometry. **(B)** B16-OVA cells were inoculated subcutaneously into LysM-Atg5^-/-^ or Atg5^flox/flox^ mice that had been adoptively transferred with OT-I cells. After tumors were established (~25 mm^2^), tumor-infiltrating lymphocytes were isolated from the tumor tissues 4 days after the final treatment of CDDP and analyzed for the expression of SIINFEKL/H-2Kb complex on TAM by flow cytometry. Representative dot plots (left) and statistical analysis (n = 4; right) are shown. (**C**) MC38 cells were injected into the spleens of Atg5^flox/flox^ or LysM-Atg5^-/-^ mice two days after intravenous administration of anti-CD8 Ab. Twenty-one days later, the mice were sacrificed and subjected to macroscopic analysis for metastatic lesions in the liver. Representative data (upper) and statistical analysis of average areas of metastatic lesions are shown (bottom). White arrows indicate metastatic tumor areas. *: *p*<0.05, ns: not significant.

Autophagy regulates multiple processes in immune responses, such as antigen processing and presentation, regulation of cytokine profiles and chemotherapy-mediated antitumor immune responses [6,30,31]. To explore the role of the autophagic pathway in myeloid cells in the regulation of effector activities, we co-cultured syngeneic wild-type CD4^+^ or CD8^+^ T cells with Atg5^flox/flox^ or LysM-Atg5^-/-^ bone marrow-derived macrophages (BMDM) loaded with dying OVA-expressing EG7 tumor cells and then analyzed the T helper cell differentiation and cytotoxic activities of T cells. LysM-Atg5^-/-^ BMDM loaded with EG7 cells resulted in the preferential induction of granzyme B^+^ CD8^+^ T cells. In contrast, lower frequencies of Foxp3^+^ regulatory T (Treg) cells were detected when co-cultured with LysM-Atg5^-/-^ compared to Atg5^flox/flox^ BMDM (Fig 5A). However, LysM-Atg5^-/-^ and Atg5^flox/flox^ BMDM had comparable ability to promote T helper cell differentiation, as shown by similar frequencies of IFN-γ^+^, IL-4^+^ or IL-17^+^ CD4^+^ T cells (Fig 5B). To further analyze the mechanisms underlying antitumor immune responses, OVA-reactive OT-I cells were adoptively transferred into LysM-Atg5^-/-^ and Atg5^flox/flox^ mice. Two days later, mice were inoculated subcutaneously with B16-OVA cells and then treated with CDDP on days 8 and 10 after tumor inoculation. Lymphocytes were isolated from the established tumors 7 days after the final treatment. CDDP treatment elicited higher infiltration of TCR-Vβ5^+^ OT-I cells into tumor tissues in LysM-Atg5^-/-^ mice compared to Atg5^flox/flox^ mice (Fig 5B). Furthermore, depletion of CD8^+^ cells with anti-CD8 monoclonal antibody accelerated the formation of metastatic foci in LysM-Atg5^-/-^ mice, whereas it had the little effect in Atg5^flox/flox^ mice (Fig 5C). Together, these results demonstrate that myeloid cell-derived autophagy contributes to the impaired presentation of tumor antigens at local tumor microenvironments.

### Impaired cross-priming of antitumor CTLs by myeloid cell-specific autophagy is dependent on TGF-β signals

**Fig 6:**
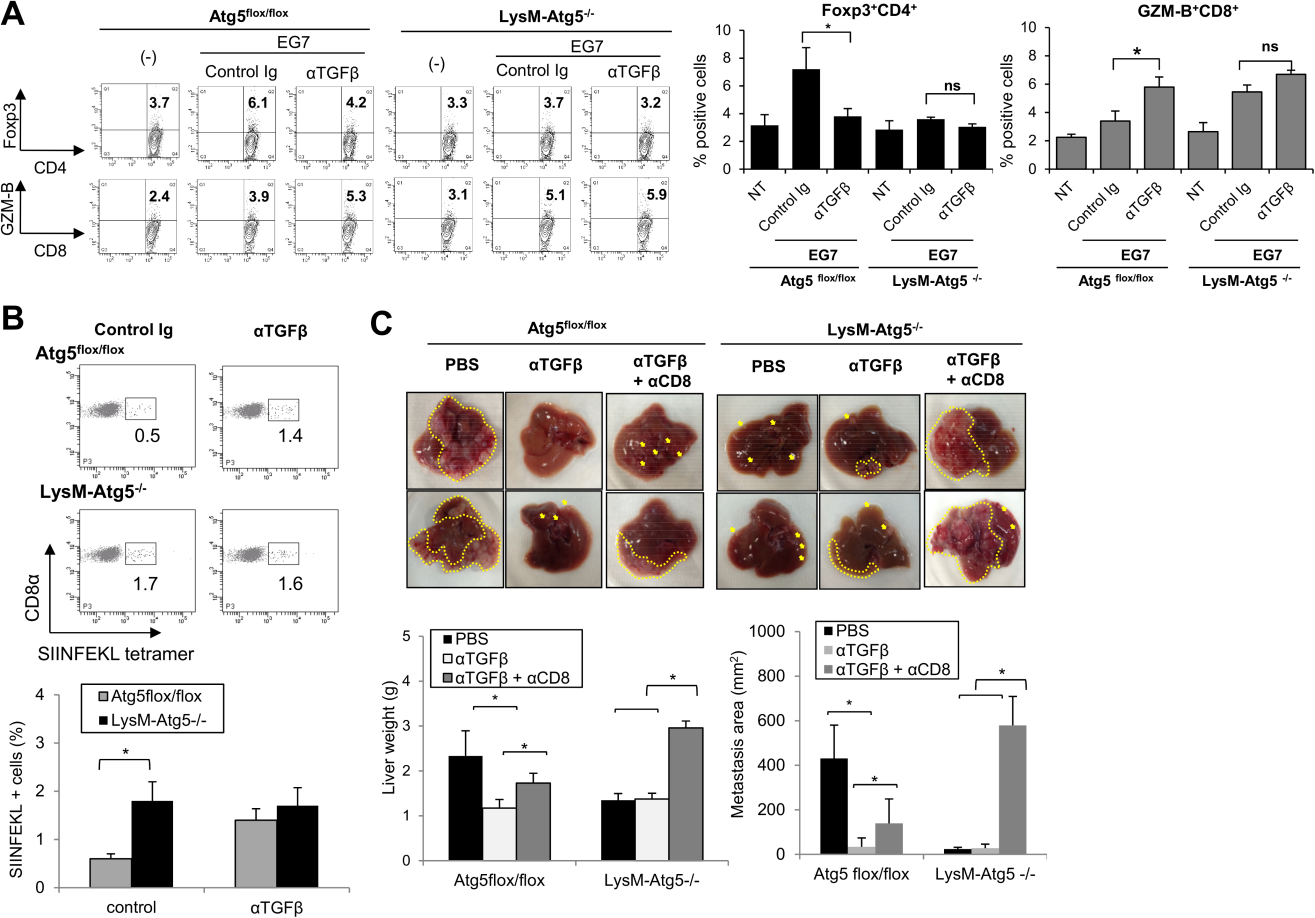
Myeloid cell autophagy suppresses antitumor immunity by TGF-β. **(A)**Atg5^flox/flox^ or LysM-Atg5^-/-^ BMDM were exposed to irradiated EG7 thymoma cells for 2 h and then co-cultured with wild-type CD4^+^ or CD8^+^ T cells in the presence of anti-TGF-β1 neutralizing mAb (1D11) or control Ig for 96 h. CD4^+^ T cells expressing Foxp3 and CD8^+^ T cells expressing granzyme-B (GZM-B) were assayed by flow cytometry. **(B)** B16-OVA cells were inoculated intravenously into Atg5^flox/flox^ or LysM-Atg5^-/-^ mice, and TAM isolated from B16-OVA tumors in metastatic lungs were co-cultured with CD8^+^ T cells for 72 hr. The expression of OVA peptide (SIINFEKL) tetramer positive cells among the CD8^+^ T cells were analyzed by flow cytometry. Representative dot plots (top) and statistical analysis (n = 3; bottom) are shown. **(C)** MC38 cells were injected into the spleen of Atg5^flox/flox^ or LysM-Atg5^-/-^ mice two days after intravenous administration of anti-CD8 depleting mAb, anti-TGF-β1 neutralizing mAb, or both. Twenty-one days later, the mice were sacrificed and subjected to macroscopic analysis for metastatic lesions in the liver. Representative data (top) and statistical analysis of liver weight and average areas of metastatic lesions (mm^2^) are shown (bottom). The tumor metastatic lesions are shown by yellow arrows. *: p<0.05, ns: not significant.

There are emerging evidences that Myeloid cell-derived TGF-β serves as critical mediator to promote tumor metastasis by suppressing IFN-ɤ and CD8+ T cell activation [23]. Moreover, TGF-β serves as a critical mediator for generating Treg cells and suppressing cytotoxic T lymphocytes (CTLs) [32–34], we next examined whether autophagy-mediated regulation of TGF-β was responsible for modulating antitumor immune responses. We found that treatment with anti-TGF-β1 neutralizing antibody largely reversed the Atg5^flox/flox^ BMDM-mediated modulation of Foxp3 expression in CD4^+^ T cells and granzyme-B expression in CD8^+^ T cells (Fig 6A). To examine whether myeloid cell-derived TGF-β regulates cross-priming of tumor antigen-specific CD8^+^ CTL in an autophagy-dependent fashion, TAM were isolated from B16-OVA-tumor-bearing Atg5^flox/flox^ or LysM-Atg5^-/-^ mice, and then used for cross-priming of OVA-specific T cells. TAMs from LysM-Atg5^-/-^ mice induced a higher percentage of CTLs reactive to an OVA-derived immunogenic peptide (SIINFEKL) compared to TAM from Atg5^flox/flox^ mice (Fig 6B). Moreover, treatment with anti-TGF-β1 Abs augmented OVA-specific responses induced by TAM from Atg5^flox/flox^ mice to levels similar to those from LysM-Atg5^-/-^ mice, implying the importance of autophagy-dependent regulation of TGF-β1 in myeloid cells in the regulation of antigen-specific CTL activation (Fig 6B).

Moreover, depletion of CD8^+^ T cells significantly attenuated the anti-metastatic effects of anti-TGF-β1 neutralizing Ab against MC38 liver tumors in the control Atg5^flox/flox^ mice, whereas it afforded LysM-Atg5^-/-^ mice with pro-metastatic ability irrespective of the treatment with anti-TGF-β1 Ab (Fig 6C). Together, these results demonstrate that myeloid autophagy negatively regulates CD8^+^ T cell-dependent antitumor immunity by promoting TGF-β production, which may be also linked with the metastatic potential of tumor cells.

### Myeloid cell-derived autophagy and tumor-derived CSF-1 coordinate the survival and differentiation of M2 macrophages in stressful tumor microenvironments

**Fig 7:**
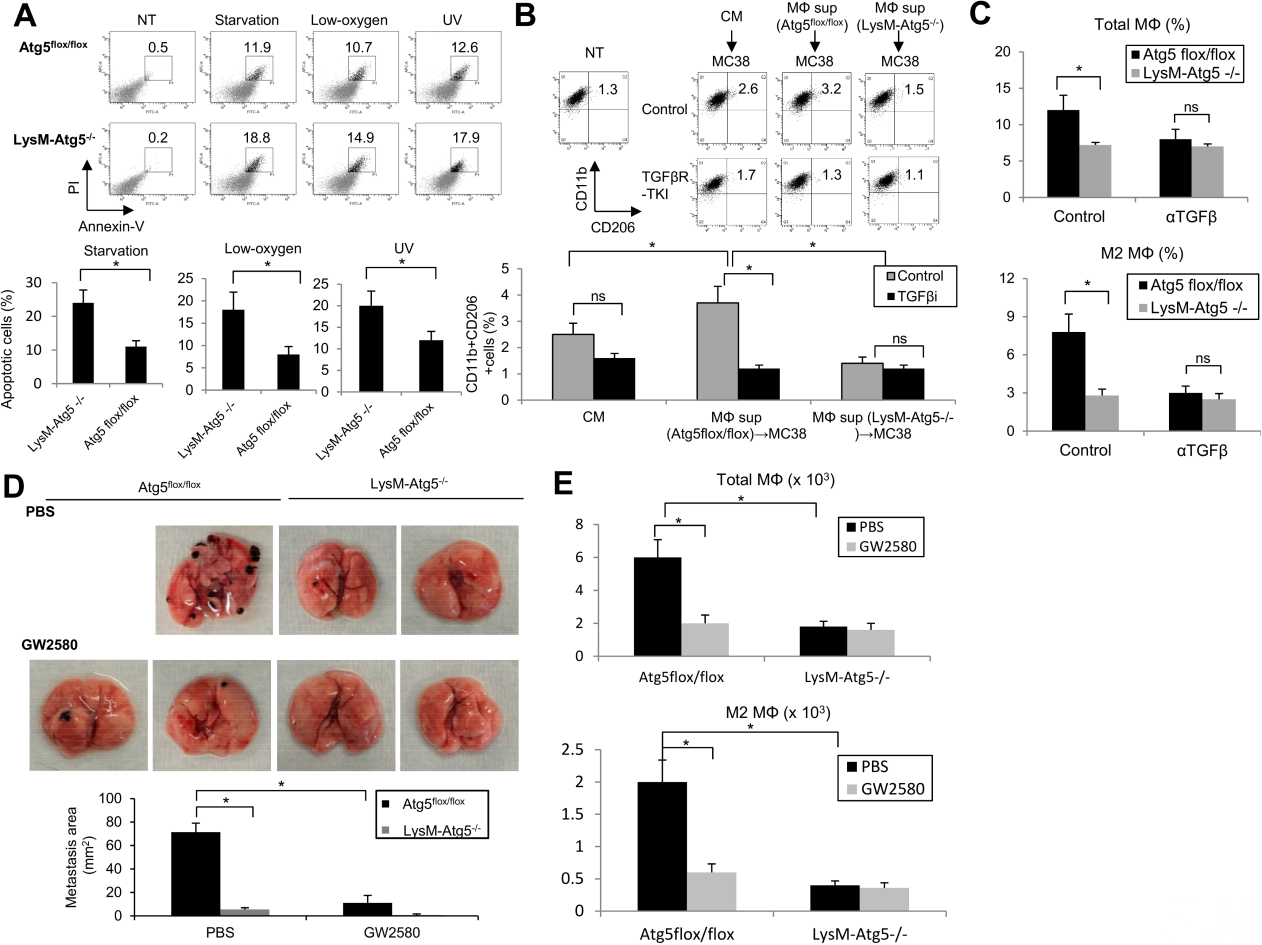
CSF-1 supports the survival and differentiation of immunosuppressive macrophages in tumors. **(A)** Atg5^flox/flox^ or LysM-Atg5^-/-^ BMDM were exposed to serum starvation for 16 h, low oxygen (1%) for 16 h, or UV light (300 J/m2) for 10 sec after 16h of culture. The cell viability was evaluated by quantifying annexin-V/PI-positive populations. Representative dot plots (upper) or statistical analysis (bottom) are shown. **(B)** MC38 cells were treated with culture medium (CM) or 50% supernatants of Atg5^flox/flox^ or LysM-Atg5^-/-^ BMDM for 72h. The supernatants of BMDM-primed MC38 cells were then used to treat wild-type BMDM in the presence of TGFβ receptor kinase inhibitor (TGFβR-KI: 10μM) or DMSO for 72 h. The expression of CD11b and CD206 was evaluated by flow cytometry. NT: untreated BMDM. **(C)** The percentages of F4/80^+^CD11b^+^macrophages, CD11b^+^CD206^high^ M2 macrophages in MC38 tumors from control Ig or anti-TGF-β-treated Atg5^flox/flox^ or LysM-Atg5^-/-^ mice were quantified by flow cytometry. **(D)** B16 cells were injected intravenously to Atg5^flox/flox^ or LysM-Atg5^-/-^ mice two days after intraperitoneal administration of GW2580 (50 mg/kg) or PBS twice per week. Twenty-one days later, the mice were sacrificed and subjected to macroscopic analysis for metastatic lesions in the lung. Representative data (top) and statistical analysis of areas of metastatic lesions (mm^2^) are shown (bottom). **(E)** The numbers of total or M2 macrophages in B16 tumors from control Ig or anti-GW2580-treated Atg5^flox/flox^ or LysM-Atg5^-/-^ mice were quantified by flow cytometry. *: p<0.05, ns: not significant.

Accumulating evidence has revealed that autophagy serves as a defense system against excess cellular stress by eliminating unfolded proteins and damaged organelles [9,10,35]. Since TME are characterized by persistent replicative stresses of tumor cells, which must adopt a strategy for surviving under conditions of hypoxia and low nutrients, we hypothesized that myeloid cells may utilize autophagic systems to survive in TME. Indeed, LysM-Atg5^-/-^ BMDM were more susceptible to various stresses, such as starvation, low oxygen or UV exposure compared to control Atg5^flox/flox^ BMDM (Fig 7A). Interestingly, tumor cells primed by LysM-Atg5^-/-^ BMDMs had a superior ability to induce the M2 markers CD206 and CD11b on wild-type BMDMs compared to tumor cells primed by Atg5^flox/flox^ BMDM. Furthermore, treatment with TGFβR-TKI abrogated the M2-priming activities of tumor cells by LysM-Atg5^-/-^ BMDM (Fig 7B). Moreover, myeloid cell-specific autophagy was responsible for the infiltration of F4/80^high^CD206^+^ M2-like macrophages in a TGF-β-dependent manner, while it had little impact on F4/80^int^CD206^low^ M1-like macrophages (Fig 7C). These findings demonstrate that myeloid cell-intrinsic autophagy contributes to survival of tumor-infiltrating myeloid cells and promotes the differentiation of immunosuppressive M2 macrophages by cooperating with autophagy-mediated production of TGF-β in stressed tumor microenvironments.

Recent studies have revealed that CSF-1 signals are critical for the survival, tumor infiltration and differentiation with immunosuppressive phenotypes of macrophages, and CSF-1 inhibitors have emerged as potent antitumor agents to prevent tumor growth and metastasis [36–38]. We therefore next examined whether pharmacological manipulation of CSF-1 impacts the tumorigenic potential of myeloid cell-derived autophagy. Interestingly, we found that CSF-1 was detected at much higher levels in tumors than in normal tissues (liver). Furthermore, treatment with CSF-1 receptor kinase inhibitor, GW2580, suppressed the lung and liver metastasis of B16 melanomas and liver metastasis of MC38 colon tumors in Atg5^flox/flox^ mice, whereas it had little effect on their metastatic potentials in LysM-Atg5^-/-^ mice (Fig 7D). Treatment with GW2580 substantially decreased the number of CD206^+^ macrophages infiltrating tumors in the control Atg5^flox/flox^ mice (Fig 7E). Taken together, these findings demonstrate that myeloid cell-derived autophagy promotes the survival and infiltration of macrophages, which further differentiate into M2-like immunosuppressive cells via tumor-derived CSF-1. This suggests that myeloid cell-derived autophagy contributes to intrinsic survival signals by acting as a defensive system against stress-prone environments, thereby leading to an increased number of TGF-β-producing pro-tumorigenic macrophages in tumor microenvironments.

### Patient-derived TAM confer tumor cells with invasive activity in an autophagy-dependent manner

**Fig 8:**
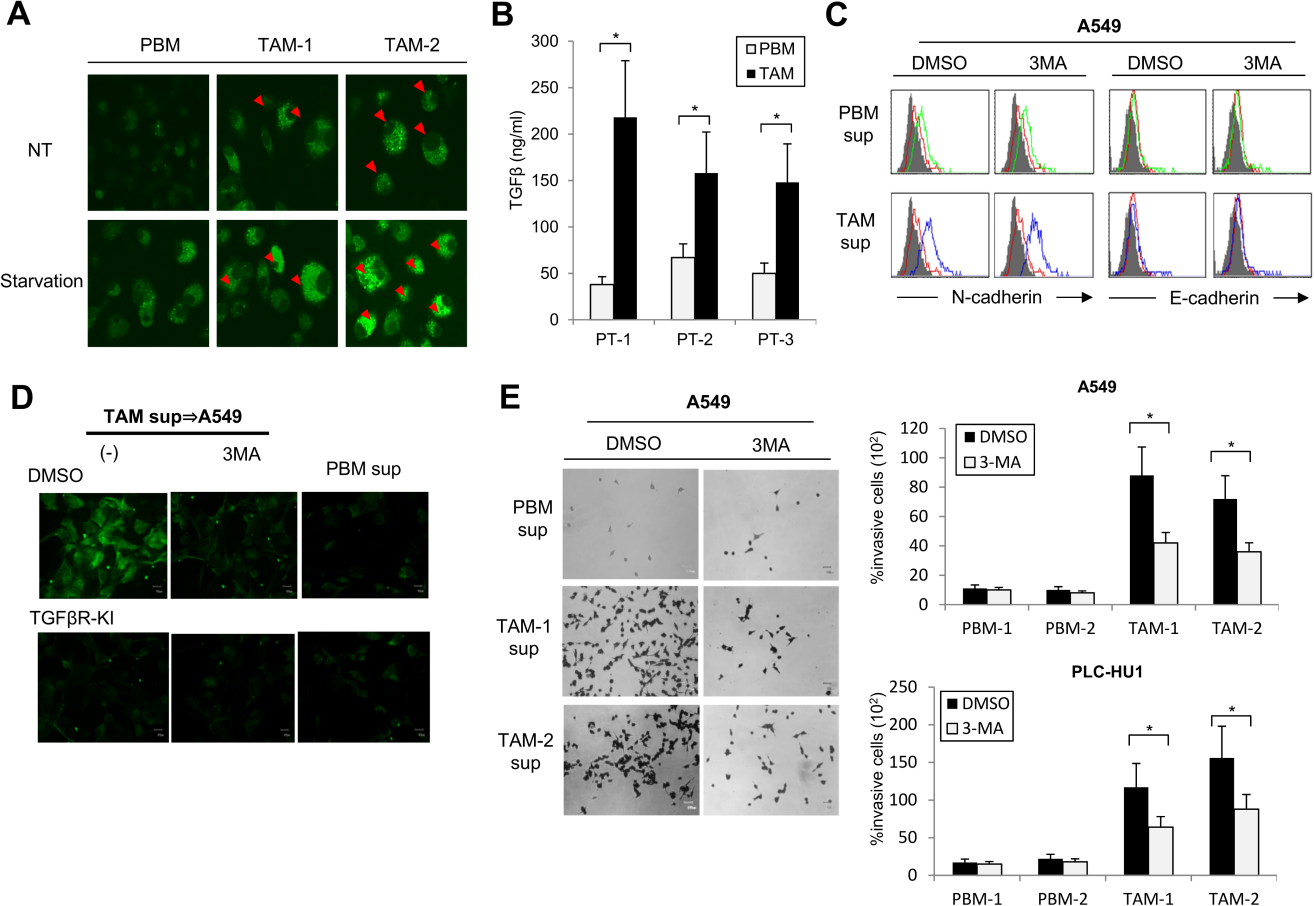
TAM-derived autophagy contributes to the invasive activities of cancer patient-derived tumor cells. **(A)** Detection of LC3 in CD11b^high^myeloid cells from tumors (TAM) or peripheral bloods (PBM) of NSCLC patients (n = 5). Red arrows indicate the cells with punctate LC3 structures. **(B)** Protein levels of TGF-β1 in culture supernatants of CD11b^high^ myeloid cells from tumors (TAM) or peripheral bloods (PBM) derived from NSCLC patients as measured by ELISA (n = 10). **(C-E)** A549 or primary NSCLC cancer cells (PLC-HU1) were cultured with supernatants of myeloid cells from tumors (TAM) or peripheral bloods (PBM) of NSCLC patients (n = 3) for 48 h in the presence of 3MA or TGFβR-KI. The indicated cells were evaluated for the expression of E-cadherin and N-cadherin by flow cytometry **(C)**, the expression of vimentin by fluorescence microscopy **(D)** and the *in vitro* invasion by Matrigel assay **(E)**. *: p<0.05, ns: not significant.

To determine whether the myeloid cell-specific autophagy-driven invasive properties of tumor cells were also operative in clinical specimens, we characterized five human CD11b^high^ myeloid cells isolated from the tumors or peripheral blood of patients with non-small cell lung cancers. We found that autophagic activities, as revealed by punctate LC3 structures, were detected at higher levels in myeloid cells isolated from tumors than in the peripheral blood of patients or healthy donors (Fig 8A). In addition, the tumor-derived CD11b^+^ myeloid cells produced TGF-β at higher levels than their peripheral blood counterparts (Fig 8B). When co-cultured with A549 or primary NSCLC tumor cells, tumor-derived myeloid cells induced an EMT phenotype, with increased levels of the mesenchymal markers N-cadherin and vimentin, as well as augmented invasive activities. Treatment with the autophagy inhibitor 3MA suppressed the induction by tumor-derived myeloid cells of EMT-associated molecules (N-cadherin and vimentin) and invasion-related phenotypes of A549 and patient-derived primary NSCLC tumor cells (PLC-HU1) (Fig 8C–8E). In accord with the importance of TGF-β in the myeloid cell autophagic systems, the treatment with TGF-βR-KI suppressed the tumor invasive activities mediated by tumor-infiltrating myeloid cells to levels similar to those of cells undergoing autophagy inhibition (Fig 8D). Taken together, these findings highlight the broad impact of myeloid cell-specific autophagy on tumorigenic activities in cancer patients.

## Discussion

**Fig 9:**
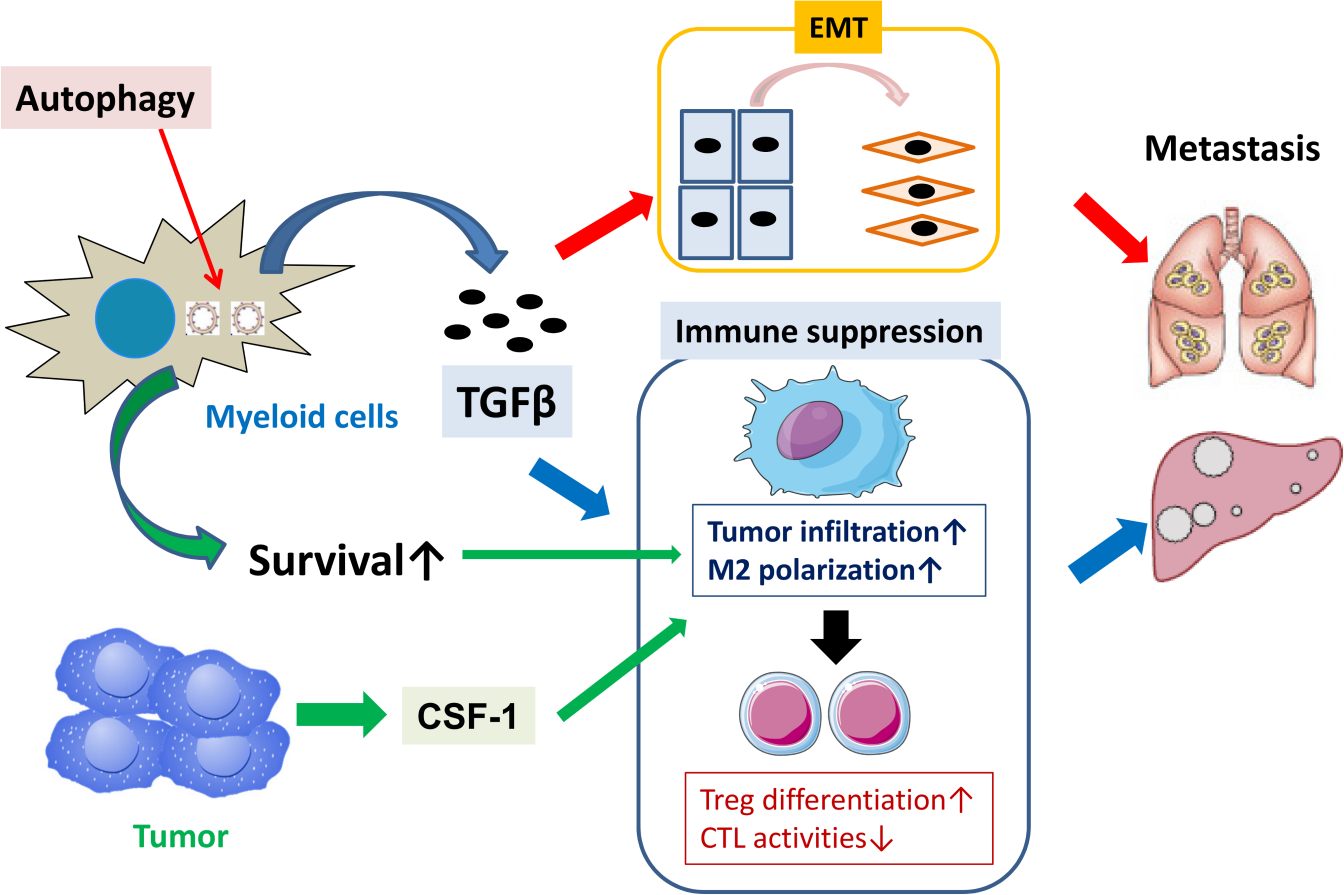
Schema of mechanism by which myeloid cell-specific autophagy promotes tumorigenesis. Myeloid cell-derived autophagy promotes tumor metastasis through TGF-β-mediated EMT, as well as activates pro-survival signals to protect myeloid cells from stress-prone tumor microenvironments in support with tumor-derived CSF-1. These dual cascade bestow the surviving tumor-infiltrating myeloid cells to further amplify pro-metastatic cascade.

Here we demonstrate that autophagic responses specifically operating in myeloid cells are key events in supporting the invasive and metastatic properties of tumor cells. As a mechanism by which myeloid cell-specific autophagy mediates tumorigenic cascades, the autophagy triggers EMT by promoting TGF-β production by myeloid cells. In accord with the importance of myeloid cell-derived TGF-β, TGF-β blockade suppressed the invasive and metastatic activities of tumors in an autophagy-dependent manner. Moreover, autophagy provides pro-survival signals to protect myeloid cells from stressful tumor microenvironments, and bestows the surviving tumor-infiltrating myeloid cells with the ability to gain pro-tumorigenic properties through tumor-derived factors such as CSF-1. These findings provide new insights into the mechanisms regulating interactions between myeloid cells and tumors (Fig 9).

Tumorigenic cells manifest deregulation of intrinsic autophagic pathways through altered signaling networks that include multiple oncogenic and tumor suppressor signals [39,40]. According to previous studies, autophagy serves as a safeguard against pro-tumorigenic metabolic and oxidative stresses, thus preventing tumor initiation. In contrast, autophagy confers a survival advantage to established tumor cells by protecting them from excessive metabolic stresses [7,9]. Thus, autophagy plays a multifaceted role in tumor initiation and progression in a cell-autonomous manner. On the other hand, it remains largely obscure whether autophagy in non-transformed stromal cells has an impact on the tumorigenesis. Our findings that myeloid cell-derived autophagy regulates metastatic potential provide a new perspective that further clarifies the functional role of autophagic pathways in the regulation of tumor progression. In accord with our findings, recent studies have also revealed the role of tumor-associated fibroblasts in supporting tumor growth by modulating the metabolic and oxidative profiles of tumor cells [41–43]. These findings raise the possibility that autophagic pathways in non-transformed stromal cells, including inflammatory cells, endothelial cells, fibroblasts as well as extracellular matrices, regulate tumorigenesis through distinct but coordinated mechanisms. In addition, previous studies revealed that genetic disruption of autophagy genes in tumor cells increased their sensitivity to diverse chemotherapeutic agents [44,45]. Thus, it is crucial to address whether myeloid cell-derived autophagy regulates the responses of tumors to anticancer therapies.

TGF-β is an important mediator in the control of tumor progression through stromal and tumor cell interactions. Herein we have demonstrated that myeloid cell-derived TGF-β contributes to tumor progression by inducing EMT in tumor cells in a paracrine manner. In addition, TGF-β modulates pro- and anti-tumor activities in TME in various ways [21–25]. Recent studies demonstrated that TGF-β activated autophagy and mediated antitumor responses in hepatocellular carcinoma cells [45]. On the other hand, TGF-β-mediated activation of EMT increased the stemness and tumorigenic activities of basal-type breast carcinoma cells through loss of fructose-1, 6-biphosphatase (FBP1) and the consequent activation of pyruvate kinase M2 [46]. Our findings further provide a new framework of TGF-β-mediated regulation of tumorigenesis, which is dependent on myeloid cell-specific autophagic pathways.

It remains unclear how autophagy regulates TGF-β production by myeloid cells. In our system, the protein levels of pro-TGF-β were decreased in LysM-Atg5^-/-^ macrophages compared to their Atg5^flox/flox^ counterparts, whereas mRNA levels were comparable in LysM-Atg5^-/-^ and Atg5^flox/flox^ macrophages. This suggests that autophagy controls TGF-β expression at the translational level. Recent studies suggest that autophagy positively regulates unconventional Golgi-ER secretory phenotypes through formation of an mTOR-autophagy spatial coupling compartment [27]. Furthermore, proinflammatory cytokines induced by autophagy-dependent secretory pathways play a critical role in increasing the metastatic potential of ras^V12^-activated melanoma cells [26]. However, a subset of immune-regulatory cytokines, such as TGF-β and IL-10, were regulated by myeloid cell-derived autophagy in our experiments, making the involvement of unconventional secretory phenotypes unlikely. Alternatively, metabolic cascades rewired as consequence of the stressed tumor microenvironments might stimulate specific secretory phenotypes in myeloid cells through coordinated activation of ER stress and autophagy, as shown in the case of senescent tumor cells [47]. Further studies are required to clarify the molecular mechanisms linking autophagic signals with the translational regulation of TGF-β in myeloid cells.

Autophagy plays a pivotal role in the regulation of innate and adaptive immune responses through multiple mechanisms. We found that myeloid cell-derived autophagy negatively regulates Th1 differentiation and cross-priming of CTL. These differences may result mainly from the distinct properties of the antigenic sources, antigen-presenting cell (APC) subsets, or microenvironments at the site of virus infection or tumors. In addition, tumors may induce different molecules and pathways, compared to infectious diseases, which influence autophagic activities in coordination with unique environmental factors. Thus, autophagy in myeloid cells may have a distinct function in immunosurveillance in different pathologic microenvironments.

Autophagy serves as a vital system to protect damaged cells from excess stress [31,35,48]. We demonstrated that myeloid cell-derived autophagy provides a safeguard for tumors by shielding them from metabolic and oxidative stresses in tumor microenvironments resulting from the reduction in oxygen and nutrients. In this sense, it is noteworthy that tumor cells have a unique intrinsic property that activates antioxidant signaling, which attenuates excess genotoxic insults, resulting in resistance to anticancer chemotherapy [49]. Moreover, CSF-1 produced at high levels from tumor tissues promotes the differentiation of CD206^+^ M2 macrophages from the surviving precursors. Even more interestingly, treatment with a CSF-1 receptor inhibitor exhibited remarkable anti-metastatic activities against tumors in control mice, whereas it showed little additive effect in autophagy-deficient mice. Thus, our present findings provide new insight into the mechanisms whereby myeloid cells support tumorigenicity through the coordinated actions of myeloid cell-intrinsic autophagy and tumor-derived myeloid-stimulating factors.

The pro-tumorigenic activities of myeloid cell-derived autophagy highlight the complex and dual roles of autophagic pathways in tumor microenvironments in the regulation of tumor progression. Although the recent development of autophagy modulators has produced significant benefits for various pathological conditions such as autoimmune and malignant disorders [50, 51], it is critical to further clarify the optimal conditions for which autophagy-targeting drugs should be used for the clinical development. Moreover, the identification of novel molecules that regulate autophagic pathways, particularly in a cell lineage-specific manner, would facilitate the development of new diagnostic and therapeutic tools useful for combating various diseases including cancer.
